# Development of two ion-selective sensors for determining benzydamine hydrochloride in the presence of its oxidative degradant across various matrices: greenness, whiteness, and blueness appraisals

**DOI:** 10.1186/s13065-025-01625-9

**Published:** 2025-09-23

**Authors:** Khadiga M. Kelani, Ragab A. M. Said, Mohammad A. El‑Dosoky, Lobna M. Abd El Halim, Ahmed R. Mohamed

**Affiliations:** 1 Pharmaceutical Analytical Chemistry Department, Faculty of Pharmacy, Cairo University, El‑Kasr El‑Aini Street, Cairo, 11562 Egypt; 2 Chemistry Department, Faculty of Pharmacy, Heliopolis University for Sustainable Development, Belbeis Desert Road, Cairo, Cairo Egypt; 3 Pharmaceutical Analytical Chemistry Department, Faculty of Pharmacy, Al-Azhar University, Nasr City, 11751 Cairo Egypt; 4 Pharmaceutical Chemistry Department, Egyptian Drug Authority, Giza, Egypt; 5 Pharmaceutical Analytical Chemistry Department, Faculty of Pharmacy, Egyptian Russian University, Badr City, 11829 Cairo Egypt

**Keywords:** Benzydamine hydrochloride, Oxidative degradation, All solidstate ion-selective electrodes, Performance limitations, Sustainability assessment

## Abstract

Two sensitive and selective ion-selective electrodes (ISEs) were developed for the determination of benzydamine hydrochloride (BNZ·HCl): a conventional polyvinyl chloride (PVC) electrode and a coated graphite all solidstate ion-selective electrode (ASS-ISE). Both sensors were constructed using an ion-pair formed between BNZ⁺ and the lipophilic anion tetraphenylborate (TPB⁻), incorporated into the sensing membranes. The sensors exhibited near-Nernstian responses with slopes of 58.09 And 57.88 mV/decade, over a Linear range of 10^–5^–10^–2^ M, and detection Limits of 5.81 × 10^–8^M and 7.41 × 10^–8^ M, respectively. They demonstrated high accuracy and precision for the determination of BNZ·HCl in pure form, pharmaceutical cream, and biological fluids, with no matrix interference. The method also proved stability-indicating, successfully detecting BNZ·HCl in the presence of its oxidative degradant. Validation was performed according to ICH guidelines, and the method showed strong environmental compatibility based on greenness, whiteness, and blueness assessments, supporting its suitability for sustainable pharmaceutical analysis.

## Introduction

**Fig. 1:**
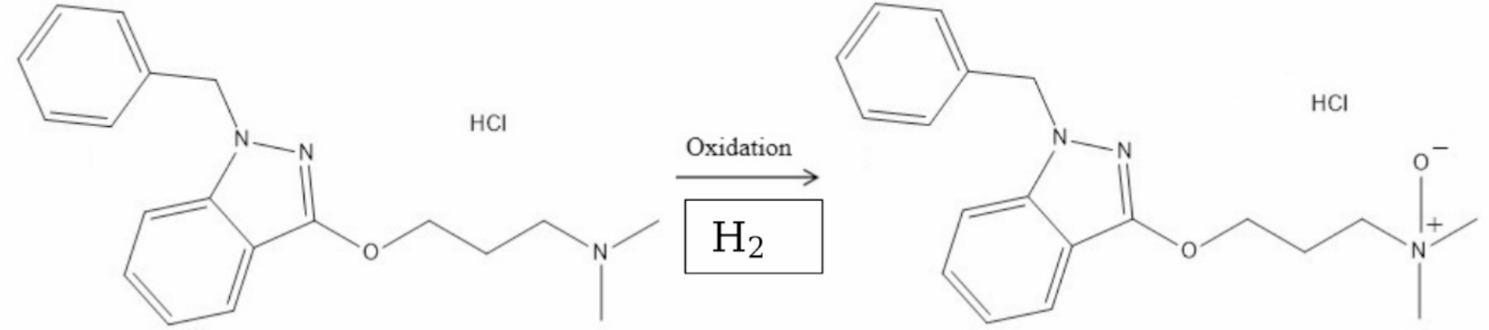
Structural formula of BNZ and its oxidative-degradant [10]

Benzydamine hydrochloride (BNZ·HCl) is a tertiary amine indazole derivative (structure shown in Fig. 1) and a nonsteroidal anti-inflammatory drug (NSAID) with local Anesthetic And analgesic properties. It is widely used in topical formulations, such as 5% (w/w) creams or gels, to treat pain and inflammation of the mouth, throat, and musculoskeletal system [1].

BNZ·HCl exerts its therapeutic effects by suppressing inflammation and inhibiting inflammatory mediators [2]. Although not inherently unstable, photodermatitis has been reported following topical use [3], indicating a potential photosensitive reaction. This may be linked to the phototransformation of BNZ into its N-oxide derivative [4], as shown in Fig. 1. Therefore, assessing the stability of BNZ under light exposure in various formulations is important.

Numerous methods have been developed for the determination of BNZ·HCl. These include reversed-phase high-performance liquid chromatography (RP-HPLC) for topical formulations [5, 6], HPLC with diode array detection for photodegradation studies [7], and fluorometric techniques for plasma monitoring following administration [8]. BNZ·HCl and its impurities have also been simultaneously quantified using HPLC in oral spray and collutory forms [9, 10].

Electrochemical approaches, including amperometric biosensors [11], piezoelectric sensors [12], and various potentiometric methods [13, 14], have gained attention due to their simplicity and cost-effectiveness. A stability-indicating UV spectrophotometric method has also been reported [15].

Ion-selective electrodes (ISEs) measure the potential difference between a selective membrane and a reference electrode, which correlates with the concentration of a target ion [16, 17]. Based on their membrane composition and design, ISEs include liquid, solid-state, glass, coated wire, enzyme-based, and gas-sensing types [18].

Potentiometric ISEs offer several advantages: rapid analysis, minimal sample preparation, low cost, portability, and broad dynamic range. They are also considered among the greenest analytical techniques due to their low environmental impact [19]. PVC-based membranes, the most common type, contain an ion pair complex embedded in a matrix of plasticizer and PVC. Alternatively, liquid membrane ISEs use ion exchangers immobilized in porous matrices to enable selective ion transport [20–22].

While ISEs are easy to use and eco-friendly, they may show limited selectivity compared to voltammetric methods, which provide higher selectivity based on electrochemical profiles but require more complex instrumentation and expertise.

The coated graphite sensor integrates a selective polymer membrane (typically PVC or similar), a plasticizer, and the ion pair complex onto a conductive graphite substrate. This design enhances conductivity, mechanical flexibility, and ease of miniaturization. Key advantages include elimination of internal solution (removing liquid junction errors), fast response, cost-efficiency, and suitability for portable or on-site applications [23].

Despite advancements in ISE technology, no prior study has reported the use of a miniaturized all-solid-state ion-selective electrode (ASS-ISE) for BNZ·HCl determination. This study introduces a novel coated graphite-based ASS-ISE, designed for compatibility with modern solid-state applications.

ISEs have been widely applied for pharmaceutical analysis [24–32], but this study marks the first use of an ASS-ISE for BNZ·HCl, representing a significant innovation in the field.

This study aimed to develop and validate two ion-selective electrodes for the determination of BNZ·HCl: a conventional PVC membrane ISE and a miniaturized coated graphite-based ASS-ISE. The goal was to establish a simple, rapid, and environmentally friendly method for BNZ·HCl analysis in pharmaceutical products, biological fluids, and in the presence of its oxidative degradant.

Both sensors were fabricated, optimized, and validated following IUPAC and ICH guidelines. Performance was assessed in standard and real samples. Additionally, the method was evaluated for environmental sustainability using greenness, whiteness, and blueness metrics, aligning the work with green analytical chemistry principles.

## Experimental details

### Materials and reagents

**Pure standard**

A pure standard of BNZ.HCl, certified by the manufacturer to contain 99.46%, was analyzed using the official USP method [33] was provided as a gift standard by EIPICO Company (Cairo, Egypt).

**Market sample**

Difflam cream 5% (Batch no. D18-2951), procured from the UK market, was labeled to contain 5% w/w of BNZ.HCl and was manufactured by Meda Pharmaceuticals Ltd., Parsonage Road, Skyway House, United Kingdom.

**Chemicals and reagents**

All chemicals and solvents used throughout the experimental procedures were of pure analytical reagent grade. Bi-distilled water Bi-distilled water was used throughout.

- Dioctyl phthalate (DOP), Tetrahydrofuran (THF), and PVC were obtained from Sigma–Aldrich, Germany.
- Sodium tetraphenylborate (Na-TPB) (Sigma–Aldrich, Germany), was prepared as a 10^−2^ M aqueous solution.
- Sodium hydroxide (El-Nasr Company, Egypt), was prepared as a 1 M aqueous solution.
- Glucose, calcium chloride, potassium chloride, sucrose, glycine, magnesium chloride, nickel chloride, and sodium chloride (El-Nasr Company, Egypt)were each prepared as 10^−3^ M aqueous solutions.
- Hydrochloric acid (El-Nasr Company, Egypt), was prepared as a 1 M aqueous solution.
- Monobasic potassium phosphate, boric acid, ethanol, methanol, sodium acetate trihydrate, and glacial acetic acid were also purchased from El-Nasr Company, Egypt. Bi-distilled water was used as a solvent for all solution preparations.

Buffers of varying pH were prepared as prescribed in USP [33]: HCl buffer (pH 2), Acetate buffer (pH 4–5.50), Phosphate buffer (pH 6–8) and Alkaline borate buffer (pH (8–10). Hydrogen peroxide (5% H_2_O_2_) was also used.

### Instruments

Measurements were carried out using a Jenway 3510 pH meter (USA) equipped with an Ag/AgCl reference electrode (model no. 924017-L03-Q11C).

A Bandelin Sonorex ultrasonic bath (model Rx 510 S) and a magnetic stirrer (Hungarian make) were also employed during the experimental procedures.

###  Stock and working solutions

A stock solution of BNZ.HCl (10^−2^ M) was prepared by dissolving 0.346 g of BNZ.HCl in 50 mL of bi-distilled water, shaking thoroughly,, And then diluting to 100 mL with bi-distilled water, Subsequently, various working standard solutions ranging from 10^−6^ M to 10^−2^ M were prepared by appropriate dilutions of the BNZ stock solution using bi- distilled water.

### Preparation of oxidative degradant

Forced oxidative degradation was carried out according to a previously reported procedure [15]. Specifically, 2 mL of a 10^−2^ M BNZ.HCl standard stock solution was added to a 100 mL flask containing 10 mL of 5% H_2_O_2_. The mixture was allowed to react for 1 h at ambient temperature, then diluted to volume with bi-distilled water. Spectrophotometric Analysis of the resulting solution was performed at 305.6 nm, using the appropriate solvent as a blank. Complete oxidation was confirmed; when the characteristic absorption peak of BNZ.HCl was no longer detectable.

### Preparation of BNZ-PVC-membrane sensor (liquid contact electrode)

#### Ion-pair complex preparation

The BNZ-tetraphenylborate associated complex was prepared by mixing 50 mL of a 10^−2^ M BNZ.HCl solution, which act as An cation with 50 mL of a 10^−2^ M sodium tetraphenylborate solution which act as Anion. The resulting solid precipitate was allowed to equilibrate with the supernatant for 6 h. The solid was then collected by filtration, thoroughly washed with bi-distilled water, And air-dried at ambient temperature for 24 h to obtain the powdered ion pair complex.

#### Sensing membrane preparation

A sensing membrane was prepared by thoroughly mixing 45 mg of dioctyl phthalate (DOP), 45 mg of polyvinyl chloride (PVC), And 10 mg of the BNZ-tetraphenylborate ion pair complex in a 5 cm diameter glass petri dish. The mixture was dissolved in 7 mL THF, and the petri dish was covered with filter paper (Whitman No. 3) to control evaporation.The setup was left undisturbed overnight at room temperature to allow for complete solvent evaporation, yielding a master membrane (sensing layer) with a thickness of approximately 0.1 mm.

#### PVC electrode assembly and conditioning

An 8-mm diameter disc was cut from the master membrane using a cork borer and affixed to the tip of a PVC electrode using THF as an adhesive. The membrane was attached to an interchangeable PVC tip, which was then clipped into the end of the glassy electrode body.

The assembled sensor was conditioned by immersion in a 10^−2^ M BNZ solution for 4 h. When not in use, the sensor was stored dry under refrigeration.

### Preparation of BNZ-coated graphite sensor (solid contact electrode)

#### Ion-pair preparation

The same procedure as in 3.3.1 was followed.

#### Sensing membrane preparation

The sensing membrane was prepared in a 5 cm diameter glass Petri dish by thoroughly mixing 45 mg of DOP, 45 mg of PVC, And 10 mg of the BNZ-tetraphenylborate complex. The resulting mixture was dissolved in 7 mL tetrahydrofuran (THF) and homogenized to ensure uniform distribution. The solvent was then allowed to evaporate slowly at ambient temperature, yielding a concentrated, oily residue suitable for coating the electrode graphite surface.

#### Coated graphite electrode assembly and conditioning

A graphite sensor was constructed using a commercially available graphite rod (3 mm diameter, 2.5 cm length). One end of the rod served as the electrical contact point, while the opposite end was repeatedly dipped into the electroactive membrane mixture until a uniform coating of the desired thickness was achieved. The coated rod was then allowed to dry completely at ambient temperature. For electrical contact, the coated end of the graphite rod was enclosed within a polytetraethylene (PTEE) tube filled with metallic mercury, into which a copper wire was inserted. Prior to use, the fabricated sensor was pre-conditioned by soaking in a 10^−2^ M BNZ solution for 6 h. When not in use, the sensor was kept dry under refrigeration.

### Measurement conditions for the suggested sensors

The reported procedure in [34] was followed.

####  Electrochemical configurations of the proposed sensors

- BNZ-PVC membrane sensor
  Internal reference electrode/internal filling solution/PVC membrane/test solution/external Ag/AgCl reference electrode.
- BNZ-coated graphite sensor:
  Graphite electrode/test solution/Ag/AgCl reference electrode.

#### Operational parameters

##### Effect of ion-pair percentage

Several membranes with varying ion-pair compositions were tested. The slopes were recorded as the ion-pair percentage increased from 4 to 12% in order to determine the slope closest to the Nernstian value and the highest achievable Nernstian slope.

##### Effect of pH and temperature

pH values ranging from 2 to 8 were tested to evaluate their effect on the response of both sensors, using 1 × 10⁻² M And 1× 10⁻³ M solutions of BEZ·HCl. The pH was adjusted using 1 M hydrochloric acid And 1 M sodium hydroxide solutions. The results of this investigation were recorded.

The effect of temperature was also studied over the range of 25 °C to 50 °C. The influence on slope values and sensor responses was examined, and the corresponding Nernstian slopes were calculated.

##### Response time

Response time refers to the duration required for the electrode to reach a stable potential. To evaluate this, three drug concentrations were selected, each ten times higher than the previous one. The results showed that the two constructed electrodes required approximately 40 s for the BNZ-PVC sensor And 50 s for the BNZ-coated graphite sensor to reach a stable potential.

##### Electrodes reversibility

To evaluate electrode reversibility, the potential was measured by sequentially decreasing the analyte concentration from higher to lower levels. This approach was used to assess the effect of concentration changes on the electrodes’ response behavior.

##### Lifetime study

The calibration slope for each electrode was measured daily and found to remain practically stable over a period of approximately three months for the PVC sensor and five months for the BNZ-coated graphite sensor.

##### Soaking time

The time required to achieve maximum And stable response - referred to as soaking time - was found to be 4 h for the BNZ-PVC membrane sensor And 6 h for the BNZ-coated graphite sensor.

##### Sensors selectivity

The potentiometric selectivity coefficients (*K*PotAB) of the proposed sensors toward various interfering substances were evaluated according to IUPAC guidelines using the separate solution method [17].

### Sensors calibration

The conditioned sensors were calibrated by transferring 25 mL of a 1 × 10⁻² M BEZ solution into 50 mL beakers. A series of BEZ solutions with varying concentrations was prepared by stepwise dilution of the 1 × 10⁻² M stock solution using bidistilled water. The electromotive force (EMF) was continuously measured for each concentration.

The fabricated sensors, in conjunction with an Ag/AgCl reference electrode, were immersed in BNZ solutions with concentrations ranging from 10^−6^ to 10^−2^ M. The solutions were gently stirred to ensure proper equilibration, and measurements were recorded once a stable potential was reached. The electromotive force (emf) values were recorded with an accuracy of ± 1 mV. Calibration curves were constructed by plotting the measured emf values against logarithmic of BEZ concentration for both sensors. The resulting calibration plots were used to calculate the Nernstain^S^ slopes. Regresian equatins were derived for subsequent measurements of unknown BEZ concentrations using the corresponding sensor.

### Preparation of laboratory mixtures

Laboratory-prepared mixtures containing different ratios of BNZ (10^−5^ M) and its oxidative degradant (ranging from 20 to 80%) were prepared and analyzed. The previously described calibration procedure was applied to determine BNZ recoveries in the presence of its oxidative degradant, thereby assessing the selectivity of the proposed method.

### Procedure validation

The proposed procedures were evaluated for linearity, limit of detection (LOD), specificity, precision, and accuracy in accordance with ICH guidelines [35].

### Applications

#### Application to pharmaceutical preparation (cream)

A 10 g sample of Difflam 5% cream was accurately weighed And transferred into a 100 mL beaker. The sample was sonicated with 20 mL of methanol for 10 min, then filtered into a 500 mL volumetric flask. The remaining residue was rinsed three times with 10 mL portions of methanol. The combined filtrate And washings were diluted to volume with bi-disteled water to obtain a cream stock solution theoretically containing 1 mg/mL of BNZ.HCl. Separately, a standard stock solution was prepared by dissolving 0.346 g of BNZ.HCl in a 500 mL volumetric flask And diluting to volume with bi-distilid water to obtain a 10^−2^ M of BNZ.HCl solution.

The general calibration procedures were then applied using appropriate volumes, covering the working concentration ranges. The BNZ.HCl content in the cream was determined using the respective regression equations derived from the calibration curves.

Additionally, the standard addition method was used by spiking the cream solution with known amounts of BNZ.HCl standard. The percentage recoveries and relative standard deviations (RSD%) were subsequently calculated to assess the accuracy and precision of the method.

#### Application to biological fluids

The reported procedure in [36] was followed.

##### Spiked serum

To 0.2 mL of serum, 1.0 mL of ethanol And 0.5 mL of 5% zinc sulfate solution were added. The resulting mixture was centrifuged at 13,000 rpm for 15 min. A clear 1 mL aliquot of the supernatant was then diluted to 14 mL with bistilled water. After deaeration for 5 min, the solution was spiked with 1 mL of a 10^−4^ M BNZ.HCl solution. The subsequent analytical steps were carried out as previously described.

##### Spiked urine

A mixture of 1.0 mL urine, 1.0 mL of 5% zinc sulfate solution, And 1.0 mL of ethanol was prepared, And the pH was adjusted to 11 by adding 0.1 mL of 1 M NaOH. The mixture was then centrifuged at 13,000 rpm for 15 min. After centrifugation, a 1 mL aliquot of the clear supernatant was diluted with 14 mL of bidistilled water And subsequently spiked with 1 mL of a 10^−4^ M BNZ.HCl solution. Subsequent steps of the procedure were carried out as previously described.

## Results and discussion

Electrochemical potentiometric techniques are robust and versatile analytical methods known for their high sensitivity, precision, and accuracy. These techniques provide a wide linear dynamic range while utilizing relatively low-cost instrumentation. Ion-selective electrodes (ISEs), a key subgroup of electrochemical sensors, convert the activity of specific ions into electrical signals. The potentiometric approach employed in ISEs enables non-destructive, low-energy analysis of electrode potentials under near-zero current conditions.

ISEs have been widely used for rapid analysis in pharmaceutical, clinical, agricultural, environmental, food, and industrial applications due to their simplicity, affordability, small size, fast response, reliability, and accuracy.

Conventional ISEs typically feature liquid-contact electrodes with an internal filling solution. However, these electrodes are subject to limitations such as membrane leaching and potential contamination. Despite these limitations,, ISEs offer significant advantages including rapid analysis, straightforward fabrication, fast response times, acceptable selectivity toward target ions, a wide linear dynamic range, suitability for online monitoring, safety, portability, and a more environmentally sustainable alternative compared to many other techniques.

Nevertheless, certain challenges remain, particularly in reducing the size of liquid-contact electrodes for sensor miniaturization and handling small sample volumes, as well as improving electrical conductivity without compromising cost-effectiveness.

To overcome these challenges, all-solid-state ion-selective electrodes (ASS-ISEs) have emerged as a promising solution. ASS-ISEs offer ease of operation, rapid measurements, low energy consumption, and enhanced potential for miniaturization. They also contribute to improved reliability, extended operational lifetime, and reduced environmental impact. For optimal function, ASS-ISEs must satisfy three critical criteria: (i) reversible ion-to-electron conduction, (ii) non-polarizable interfaces with high capacitance, and (iii) absence of side reactions [37].

This study focuses on developing two types of ion-selective electrodes (ISEs) for the determination of BNZ.HCl: a PVC membrane sensor and a coated graphite sensor, which also serves as an ASS-ISE. The method exploits the cationic nature of BNZ.HCl (BEZ+), which interacts with an anionic site (TPB-) of large lipophilic additive sodium tetraphenylborate (Na-TPB) to form a water-insoluble ion pair (BEZ–TPB) via precipitation. The resulting precipitate possesses a low solubility product, suitable particle size distribution, and physical compatibility with the electrode matrix. The high selectivity of both sensors enables accurate determination of the drug even in the presence of oxidative degradants, excipients, and biological matrice.

### Performance features of the suggested sensors

The performance of the proposed sensors was evaluated following IUPAC recommendations [17]. Calibrations were performed by immersing the electrodes alongside an Ag/AgCl reference electrode in BNZ.HCl solutions over a concentration range of 10^−5^M–10^−2^M. Potential measurements obtained from serial analyses of standard BNZ.HCl solutions showed consistent responses both within the same day and over a two-week period, with variations not exceeding ± 1 mV/decade. Key performance characteristics of the proposed sensors are summarized in Table 1.

**Table 1:** Key performance characteristics of the proposed sensors

Parameter		PVC sensor	Coated graphite sensor
- Regression equation - Slope (b) - Intercept (a)		y ^a^ = b x ^b^ + a 58.09 213.44	y ^a^ = b x ^b^ + a 57.88 255.19
Coefficient of determination (r^2^)		0.9995	0.9996
Linear range (M)		10^−5^ – 10^−2^	10^−5^ – 10^−2^
Working pH range		2–8	
Response time		40 s	50 s
LOD (M)		5.81 × 10^−8^	7.41 × 10^−8^
Accuracy (%R) ^c^		98.07	98.61
*Precision (% RSD) * ^c^			
Repeatability		0.518	0.438
Intermediate precision		0.824	0.733

^a^The recorded sensor potential

^b^The molar concentration of the drug

^c^Values for 3 determinations of 3 different concentrations

### Optimization of the proposed sensors

#### Influence of ion-pair percent

The ion-pair complex is a crucial component in ion-selective electrodes (ISEs), acting as the electroactive material responsible for the selective recognition and detection of the target ion by the sensor. Its percentage composition within the membrane significantly affects the sensor’s performance, including sensitivity, selectivity, and response stability.

##### BEZ-PVC membrane sensor

The primary constituents of the PVC membrane sensor include PVC, an ion complex, and a plasticizer. Optimal sensor performance depends on maintaining appropriate weight ratios among the ion- pair, plasticizer (DOP), and PVC during membrane preparation. BEZ-tetraphenylborate was synthesized and evaluated as a sensor modifier. The study involved varying the weight% of the ion- pair, while maintaining the PVC-to-plasticizer ratio constant at 1:1, as summarized in Table 2. Notably, the sensor containing 10% (w/w) BEZ-tetraphenylborate exhibited a near-Nernstian slope of 58.09 mV/decade.

**Table 2:** Optimization of the membrane composition (w/w%) of the PVC membrane and coated graphite sensors*

Sensor	Composition % (w/w)			Linear range (M)	Slope (mV/decade)	r^2^
	BNZ-TPB	PVC	DOP			
PVC membrane sensor	4	48	48	1 × 10^−5^ – 1 × 10^−2^	54.31	0.9984
	8	46	46	1 × 10^−5^ – 1 × 10^−2^	55.42	0.9991
	**10**	**45**	**45**	**1 × 10**^**−5**^ **– 1 × 10**^**−2**^	**58.09**	**0.9995**
	12	44	44	1 × 10^−5^ – 1 × 10^−2^	56.77	0.9995
Coated graphite sensor	4	48	48	1 × 10^−5^ – 1 × 10^−2^	53.68	0.9989
	8	46	46	1 × 10^−5^ – 1 × 10^−2^	55.05	0.9993
	**10**	**45**	**45**	**1 × 10**^**−5**^ **– 1 × 10**^**−2**^	**57.88**	**0.9996**
	12	44	44	1 × 10^−5^ – 1 × 10^−2^	56.07	0.9994

*BNZ-TPB* benzydamine-tetraphenylborate

*The bold values reflect the appropriate composition % (w/w) of the ion pair, plasticizer (DOP) and PVC, that exhibited the near-Nernstian slopes of both sensors

##### BNZ-boated graphite sensor

BNZ-tetraphenylborate, the ion-pair complex, was synthesized and evaluated as a modifying agent for the sensor. The evaluation involved adjusting the weight% of the complex while maintaining a fixed 1:1 ratio between the PVC and plasticizer, as detailed in Table 2. The sensor containing 10% (w/w) BNZ-tetraphenylborate exhibited a slope of 57.88 mV/decade.

#### Efferct of soaking time

Freshly prepared sensors should be soaked to activate the membrane’s surface toform a thin gel layer where exchange occurs. The proposed sensors were immersed in a 1 × 10^−2^ M BNZ.HCl solution, And calibration curves were recorded at various time intervals ranging from 0 to 12 h. Measurements continued until significant deviations from the expected Nernstian slopes were observed. As summarized in Table 3, the optimal soaking times were found to be 4 h for the BNZ-PVC sensor And 6 h for the BNZ- coated graphite sensor.

**Table 3:** Effect of soaking time on the proposed sensors*

Soaking time/hour	PVC sensor	Coated graphite sensor
	Slope (mV/decade)	
0	48.19	47.89
2	53.62	49.14
**4**	**58.09**	52.26
**6**	56.13	**57.88**
8	54.38	55.43
10	52.11	53.77
12	50.87	52.06

*The bold values reflect the maximum soaking time/hour that exhibited the near-Nernstian slopes of both sensors

#### Effect of pH and temperature

**Fig. 2:**
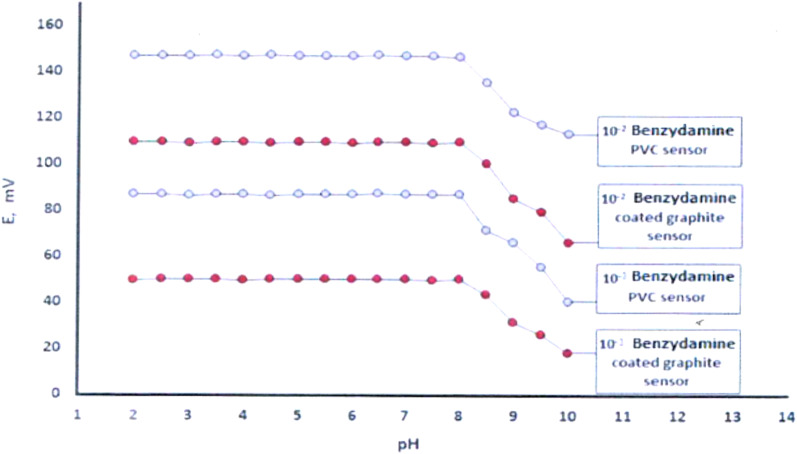
Effect of pH on the response of BNZ using PVC membrane and coated graphite sensors

To determine the optimal working pH range, the stability of potential readings was evaluated across a pH range of 2 to 8. Measurements were performed using 1 × 10^−2^ M And 1× 10^−3^ M BNZ.HCl solutions, with potential recorded at each pH value. Representative curves illustrating the effect of pH on both sensors are presented in Fig. 2. Both the BNZ-PVC And BNZ-coated graphite sensors exhibited stable potentials within the pH range of 2 to 8. The electrodes’ slopes remained within the acceptable range (± 10% of the slope) without significant reduction.

The sensor responses And the slopes also remained constant within the temperature range of 25–50 °C, indicating good thermal stability.

#### Sensors’ response time

For analytical applications, the response time of the prepared sensors is of critical importance. Response time is defined as the average time required to reach a stable potential (response) within ± 1.00 mV of the final equilibrium after sequential immersions in BNZ.HCl solutions with tenfold concentration increments, was evaluated. As detailed in Table 1, the BNZ-PVC membrane sensor exhibited stable responses within 40 s, while the BNZ-coated graphite sensor reached stability within 50 s.

#### Reversability and lifetime

Reversibility was also investigated, with no change observed in the reversed response, indicating stable sensor performance. The operational Lifetimes were determined to be 3 months for the PVC membrane electrode And 5 months for the coated graphite electrode. This demonstrates that the ASS-ISE offers a long operational lifespan.

#### Sensors selectivity

The potential interference from related compounds on the sensors’ response to the target drug (BNZ.HCl) was evaluated. Independent potential measurements were carried outusing separate 10^−3^ M solutions of BNZ.HCL And the potential interfering ions. Selectivity coefficients were then calculated using the following mathematical equation [38]:

$$K^{pot}_{Drug\, J ^{+z}}=\frac{{E}_{2}-{E}_{1}}{S}+\text{log} [drug]-\text{log}[{{J}^{+z}}]^{1/2}$$

In the equation, E_1_ and E_2_ represent each electrode response measured for a 10^−3^ M solution of BNZ.HCl and interferent ion [J^+z^], respectively, while S denotes the slope of the calibration curve. The tested interfering materials included magnesium chloride, calcium chloride, potassium chloride, nickel chloride, sodium chloride, glucose, sucrose, glycine, urea, and the oxidative degradant of BNZ.HCl. The selectivity coefficients calculated for the developed sensors are presented in Table 4. developed sensors. Table 5 shows the ability of all sensors to accurately determine BNZ.HCL without interference from its oxidative degradant at various ratios.

**Table 4:** Selectivity coefficients of the proposed sensors using the separated solution method

Interferent	− log $\:{K}_{Drug{J}^{+z}\:}^{pot}$ of PVC membrane sensor	− log $\:{K}_{Drug{J}^{+z}\:}^{pot}$ of coated graphite sensor
Oxidative-degradant	1.010	1.098
Calcium chloride	1.004	1.059
Magnesium chloride	1.002	1.019
Sodium chloride	1.007	1.091
Nickel chloride	1.014	1.068
Glucose	1.029	1.081
Urea	1.088	1.077
Glycine	1.062	1.039
Sucrose	1.084	1.053

**Table 5:** Determination of BNZ in the presence of oxidative-degradant by the proposed sensors

Intact drug (M)	Degradant%	Recovery %^a^	
		PVC sensor	Coated graphite sensor
×110^−5^ M	80	95.59	96.01
×110^−4^ M	40	97.01	98.72
×110^−5^ M	20	99.55	98.52
×110^−4^ M	10	99.97	100.07

^a^Mean of three determinations

### Development and optimization of sability-indicating methods

Given the known photosensitivity of BNZ·HCl, forced oxidative degradation was performed using 5% hydrogen peroxide (H₂O₂) to simulate stress conditions in line with FDA guidelines. Direct exposure to UV light resulted in negligible degradation, confirming prior findings [7, 10, 15]. Complete oxidative degradation was achieved after one hour of H₂O₂ treatment at ambient temperature. The drug was accurately quantified in the presence of 10–80% of its degradant, as summarized in Table 5.

While conventional ISEs with internal filling solutions suffer from limitations such as membrane leaching and contamination, the electrodes developed in this study offer significant advantages. These include fast response times, simple fabrication, a wide linear dynamic range, satisfactory selectivity, portability, and improved environmental compatibility, making them well-suited for practical pharmaceutical analysis and real-time monitoring.

### Method validation

#### Linear range

**Fig. 3:**
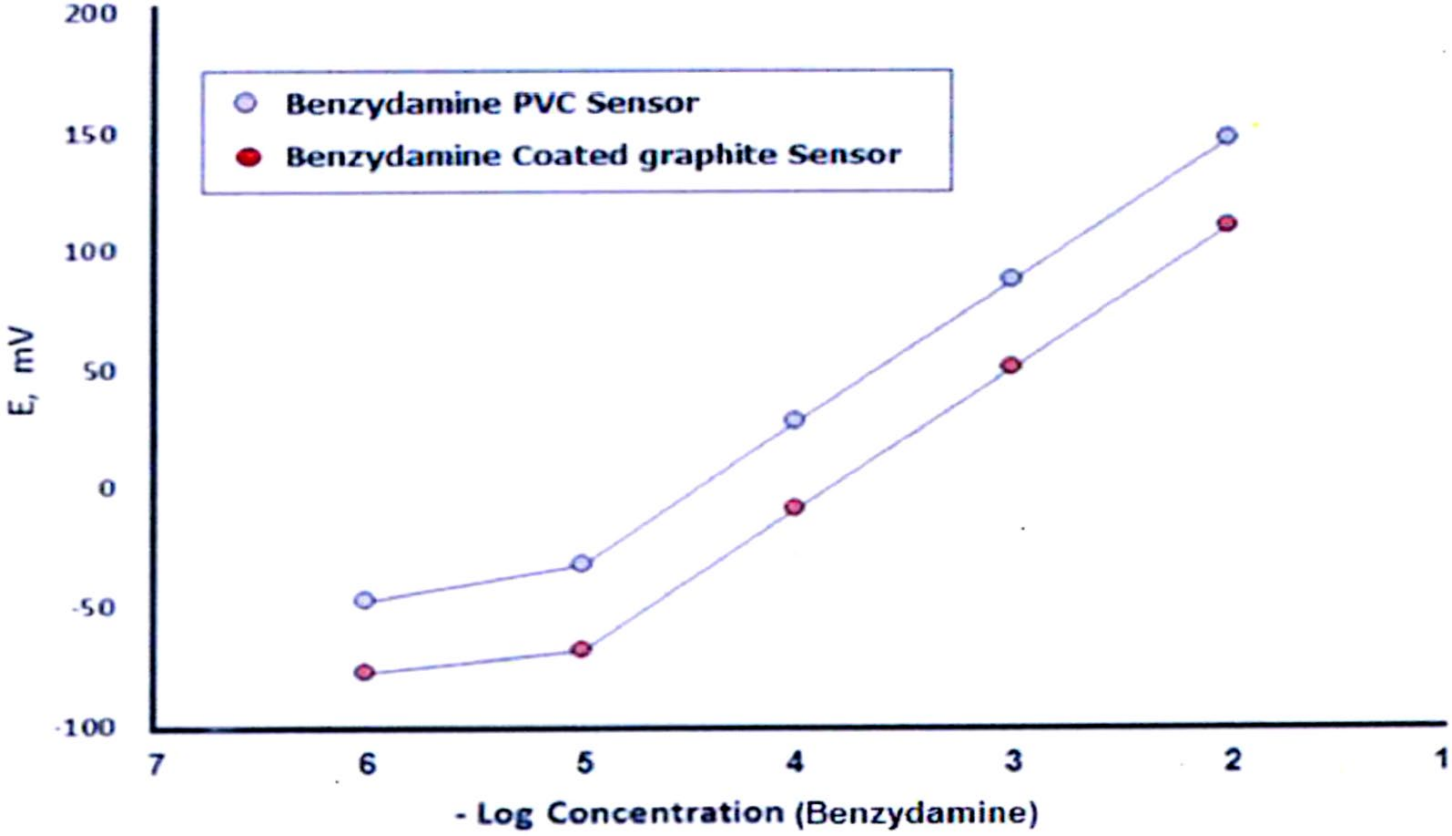
Profile of the potential in mV/− Log molar concentration of BNZ using PVC membrane and coated graphite sensors

Under the established experimental conditions, calibration curves for each proposed sensor were generated by plotting the measured sensor potentials against the negative logarithm (− Log) of BNZ.HCL concentrations. The resulting regression plots demonstrated Linearity over the concentration range of 10^−5^M–10^−2^ M for both sensors, as illustrated in Fig. 3.

#### Limit of detection

The limit of detection (LOD) was estimated by identifying the intersection point of the extrapolated linear segments in Fig. 3. The LOD values were found to be 5.81 × 10^−8^ M for the PVC sensor And 7.41 × 10^−8^ M for the graphite sensor. These low LOD values indicate a high degree of sensitivity of the proposed sensors. The results are summarized in Table 1.

#### Accuracy and precision

**Accuracy**

The accuracy of the developed method, expressed as the mean recovery percentage (R%), was evaluated by performing triplicate analyses at three BNZ.HCL concentration levels within the linear range (10^−2^ M − 10^−5^ M). The results, presented in Table 1, demonstrate the acceptable accuracy of the proposed methodology.

**Precision**

The precision of the developed methodology, expressed as the percentage relative standard deviation (%RSD), was assessed through triplicate analyses at three BNZ concentration levels (10^−2^ M − 10^−5^ M) within the linear range. Repeatability was evaluated by performing measurements within a single day, while intermediate precision was assessed over three consecutive days. The low % RSD values, presented in Table 1, confirm the high precision of the proposed method.

#### Specificity

The specificity of the proposed potentiometric method was demonstrated by its ability to accurately assay the drug in its pharmaceutical formulation without interference from common preservatives or excipients. The high specificity achieved by the developed sensors is summarized in Table 6.

**Table 6:** Determination of BNZ in cream formulation; application of standard addition technique

Cream formulation (B.no. D18-2951)	Found^a^ ± SD	Added BNZ	Recovery^b^ of added BNZ
1 × 10^−4^ M	98.12 ± 1.446	1 × 10^−5^ M	99.80
		5 × 10^−4^ M	99.01
		1 × 10^−4^ M	98.34
Mean ± RSD %		99.05 ± 0.74	

^a^Mean of three determinations

^b^Average of three replicates experiments

#### Robustness

The robustness of the proposed method was demonstrated by the stability of the measured potential (mV) despite deliberate minor alterations in experimental conditions. Variables assessed included the time (in seconds) before each measurement (10 s ± 5 s) and the electrode soaking time (4–6 h ± 30 min). These intentional changes had no significant effect on the mV readings for BNZ.HCl, indicating the method’s reliability And consistency under typical operational conditions. Additionally, the effect of pH was studied at different values of 4, 4.5, 5, 5.5, 6.0, 6.5, And 7.0 using a 1.0 × 10⁻^3^mol L⁻¹ BNZ·HCl solution, with no observable change in electrode response across this pH range.

### Statistical comparison with reported method

The analytical performance of the proposed method was benchmarked against a previously reported method [9] through statistical comparison. The absence of significant differences, confirmed by t-test And F-test Analyses at a 95% confidence level, validates the acceptable accuracy and precision of the recommended methodology for quantifying BNZ.HCl in its pharmaceutical formulations (Table 7).

**Table 7:** Statistical comparison between the proposed method and the reported method

Parameters	Sensors		Reported method [19]
	PVC	Coated graphite	
N^a^	5		5
Mean	98.73	99.08	100.68
Variance	0.506	0.419	0.595
%RSD	0.702	0.641	0.766
Student’s t-test (2.31)^b^	1.481	0.977	–
F- value (6.39)^b^	1.177	1.421	–

^a^Number of measurements

^b^The values in parenthesis are tabulated values of “t” and “F” at (*P* = 0.05)

### Applications

#### Application to pharmaceutical formulation (cream)

All proposed sensors were tested for selective determination of BNZ.HCl in Difflam 5%. cream The results demonstrated satisfactory performance, closely aligning with the labeled content. Recoveries obtained using the standard addition technique for known amounts of BNZ.HCl spiked into the pharmaceutical formulation (cream) ranged from 98.34 to 99.80%, as presented in Table 6.

#### Application to biological fluids

The studies confirm that BNZ is systemically absorbed. The results obtained from spiked biological samples—serum and urine samples [39–41]—are presented in Table 8. These findings indicate that the proposed method is suitable for the analysis of BNZ.HCl in biological fluids, with no significant interference from the sample matrices.

**Table 8:** Application of the proposed potentiometric method using coated graphite sensor for the determination of bnz.hcl in biological fluid [36]

Spiked intact drug (M)	Found %^a^	
	Plasma sample	Urine sample
1 × 110^−5^ M 1 × 10^−4^ M	96.59	95.71
	97.41	96.42
	98.55	97.52
	98.97	97 0.07
Mean ± RSD %	97.98 ± 1.12	96.9 ± 0.94^a^

^a^Mean of three determinations

### Comparison with published methods

Numerous potentiometric methods using ion-selective electrodes (ISEs) have been reported for the determination of Benzydamine HCl in pure form, pharmaceutical formulations, or saliva samples. However, only our method successfully determines BNZ.HCl in the presence of its oxidative degradant using a fabricated all-solid-state ion-selective electrode (ASS-ISE). This solid, miniaturized, coated graphite sensor offers several advantages: the graphite rod is highly conductive, compact, cost-effective, easy to fabricate, and has a long operational lifetime.

In contrast, other reported methods have notable limitations. For example, the screen-printed ISE [13] is expensive, not widely available, disposable, and has a short lifespan with reduced stability. Similarly, the silver wire-coated electrode [39] is prone to oxidation, requires polishing before each use, and also suffers from limited stability and a short operational lifespan.

Compared to other instrumental methods such as spectrophotometric or chromatographic techniques, which offer high sensitivity and selectivity, potentiometric methods are more cost-effective, require no solvents and utilize simpler instrumentation, and are more environmentally friendly.

Table 9 summarizes the key differences between the proposed method and previously reported UV [15], electrochemical [11, 12, 39], and chromatographic techniques, including HPLC and TLC [6, 7, 10, 42]. It highlights the effect of oxidative degradants on the percent recovery of Benzydamine HCl. The results demonstrate that the coated graphite solid-state sensor offers significant advantages over earlier methods, particularly in terms of accuracy and robustness against degradation.

**Table 9:** Comparison of different reported methods and the proposed potentiometric method for BNZ.HCl determination

Items	UV method [15]	Chromatographic methods		Electrochemical methods		Ion-selective electrodes	
		HPLC [6, 7, 10]	TLC [42]	Method [11]	Method [12, 39]	PVC	Coated graphite
%Recovery	88%	98.25	Identification	Separation and identification	100.20	98.73	99.08
Greenness	+VE	−Ve	−Ve	+VE	+VE	+VE	+VE
Sustainability	−Ve	−Ve	−Ve	−Ve	−Ve	−VE	+VE
Portability	−Ve	−Ve	−Ve	−Ve	−Ve	−VE	+VE
Miniaturization	−Ve	−Ve	−Ve	−Ve	−Ve	−VE	+VE
Cost-effective	−Ve	−Ve	−Ve	−Ve	−Ve	+VE	+VE

+VE sign means applicable

− VE sign means not applicable

While potentiometric sensors offer notable advantages such as simplicity, low cost, portability, and real-time monitoring without complex instrumentation [43, 44], they may exhibit lower selectivity under certain analytical conditions compared to voltammetric methods [44, 45].

### Limitations

While the developed ion-selective electrodes (ISEs), including PVC membrane and coated graphite electrodes, demonstrated satisfactory performance in terms of sensitivity, selectivity, and robustness, several inherent methodological limitations remain. One key challenge is the potential for electrode contamination, particularly in complex sample matrices. For instance, PVC membranes can experience plasticizer leaching and surface fouling from organic compounds, while coated graphite electrodes may suffer from baseline drift due to surface adsorption and redox interference.

Selectivity is another notable limitation. Although optimized ionophores were used, cross-sensitivity to ions with similar charge or size, as well as matrix effects in high ionic strength samples, can affect accuracy. Additionally, both types of electrodes may exhibit potential drift, limited lifespan due to membrane degradation, and batch-to-batch variability in electrode preparation.

Despite these limitations, the sensors developed in this study were optimized for key parameters such as pH, temperature, response time, and selectivity. These optimizations help mitigate some of the challenges and support the practical utility of ISEs in applications requiring fast, cost-effective, and environmentally friendly analytical methods.

### Sustainability assessments (blueness, greenness, and whiteness appraisals)

With the growing demand for eco-friendly, low-waste, and resource-efficient methods, greenness assessment tools have become essential for evaluating the environmental impact of analytical procedures.

Electrochemical techniques inherently align with green chemistry principles by minimizing reagent use, reducing energy consumption, and enabling in situ, real-time analysis—often without extensive sample preparation or hazardous solvents. However, as new sensor technologies and electrode materials emerge, it becomes increasingly important to systematically assess their environmental footprints.

To address this need, various greenness evaluation tools have been developed and adapted for electroanalytical applications. Metrics such as the Analytical Eco-Scale, Green Analytical Procedure Index (GAPI), and AGREE (Analytical GREEnness Metric) offer structured frameworks for assessing ecological and safety aspects of analytical methods. These tools consider multiple criteria, including the toxicity of reagents, waste generation, energy consumption, and procedural complexity. Their application provides a transparent and quantitative means of comparing alternative methods and guiding researchers toward more sustainable analytical practices.

Furthermore, the integration of these greenness metrics into method validation processes encourages the design of environmentally responsible electrochemical sensors and potentiometric devices. By prioritizing non-toxic materials, solvent-free or aqueous systems, and recyclable components, electrochemists contribute not only to scientific advancement but also to the broader goals of environmental stewardship and regulatory compliance.

Furthermore, integrating these greenness metrics into method validation encourages the design of environmentally responsible electrochemical sensors and potentiometric devices. By prioritizing non-toxic materials, solvent-free or aqueous systems, and recyclable components, electrochemists advance both scientific innovation and the broader goals of environmental stewardship and regulatory compliance.

**Fig. 4:**
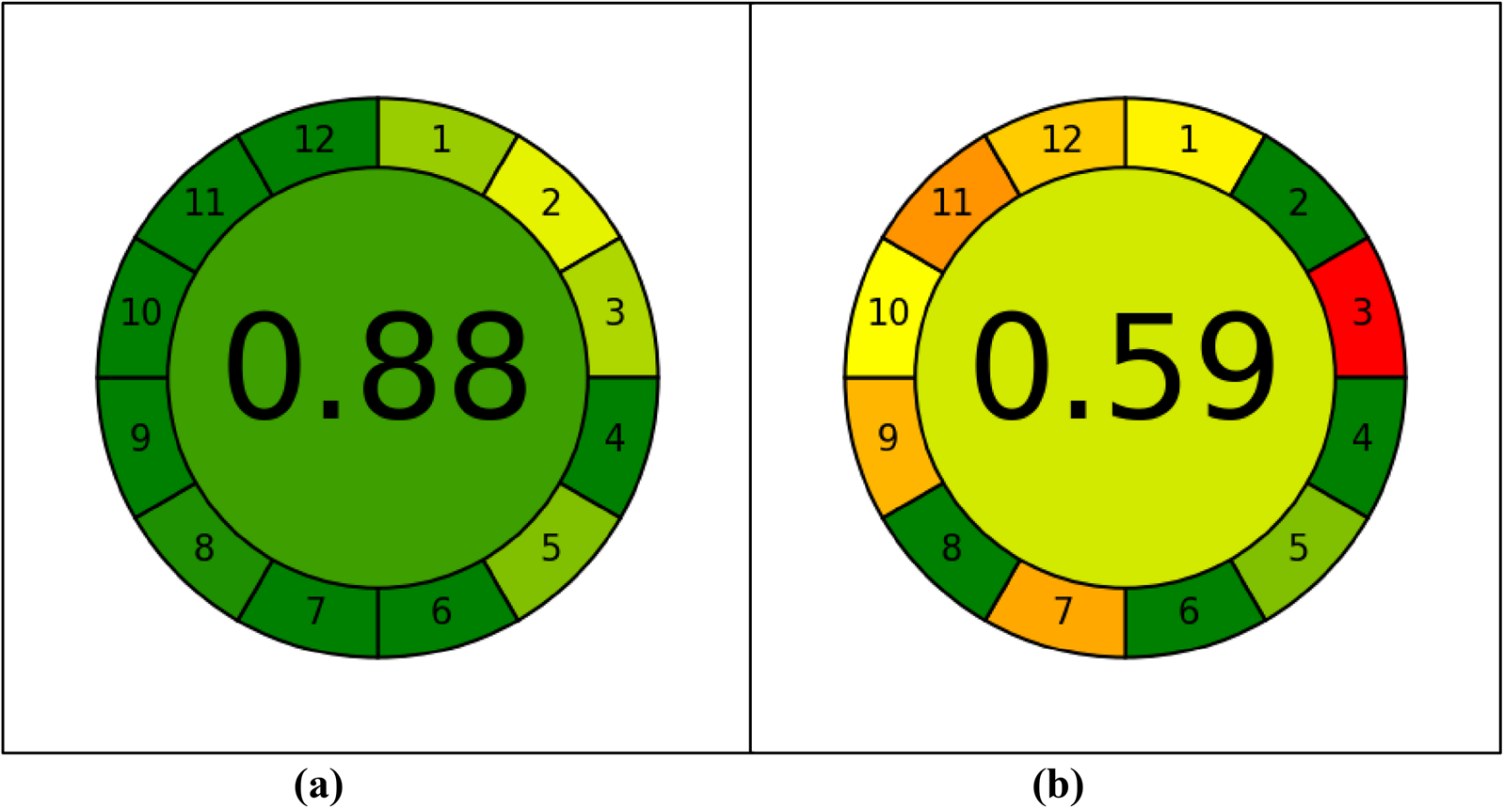
Greenness profiles of the suggested (**a**) and reported [9] (**b**) techniques using the AGREE tool

The AGREE open-source program generates a 12-segment circular pictogram designed to evaluate green analytical chemistry (GAC) practices. Each segment corresponds to one of the 12 GAC principles with colors ranging from green for minimal impact to red for significant impact), providing both qualitative and quantitative information about greenness of a method [46]. The overall AGREE score, displayed at the center of the pictogram, summarizes the method’s overall environmental performance. The AGREE pictograms (Fig. 4) and their associated overall scores, both for the method presented here and those previously reported [47], clearly demonstrates the superior greenness of the techniques developed in this study.

**Fig. 5:**
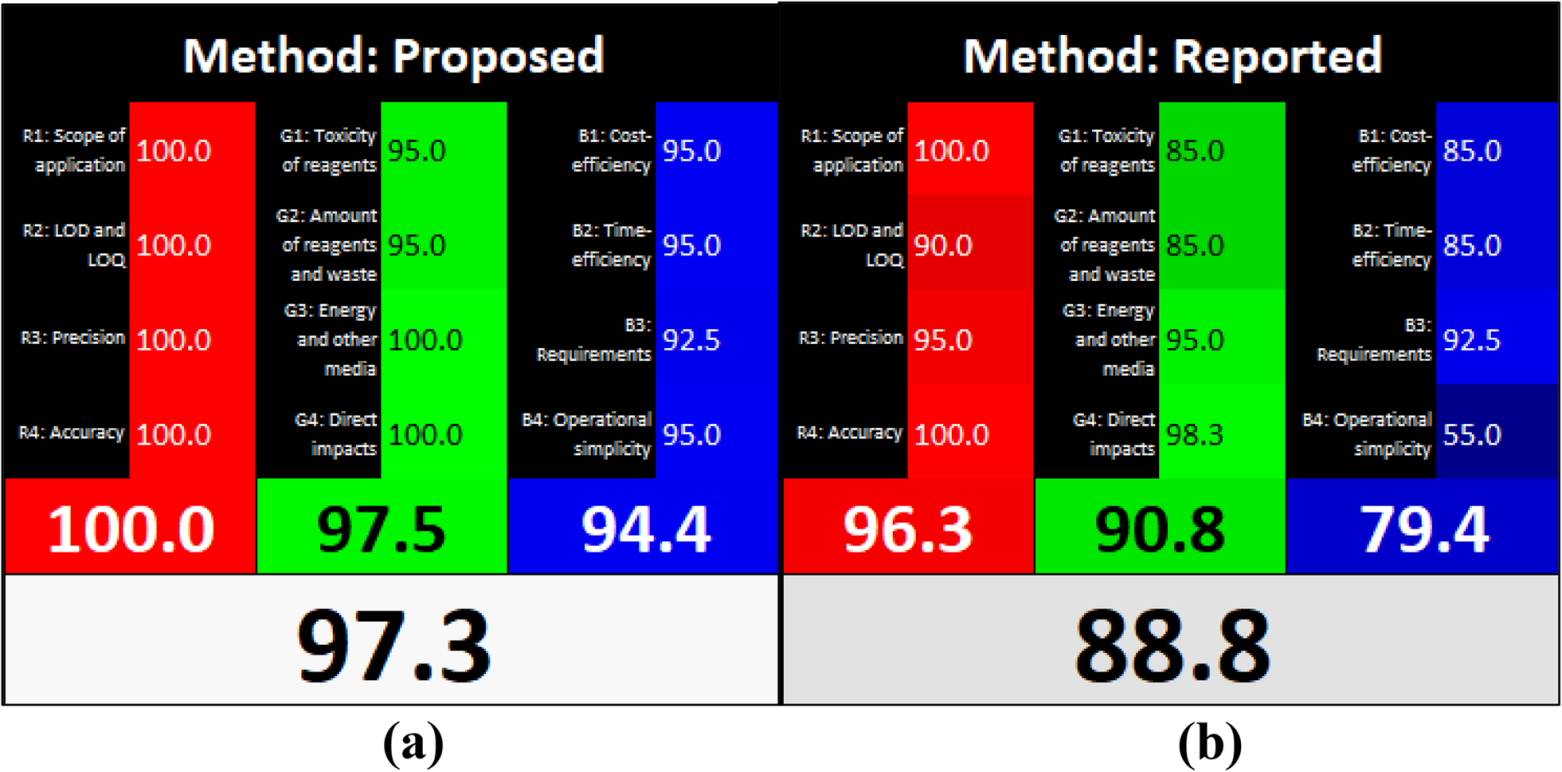
Whiteness appraisal of the proposed and reported [26] methods using the RGB12 algorithm

The proposed and previously reported techniques were evaluated for their “whiteness” using the RGB12 model [46, 47]. This model utilizes twelve Excel based algorithms grouped into blue, green, and red categories, each representing different aspects of method performance. As illustrated in Fig. 5,the proposed technique achieved superior “whiteness” scores compared to the previously reported method [9], underscoring its enhanced overall analytical performance.

**Fig. 6:**
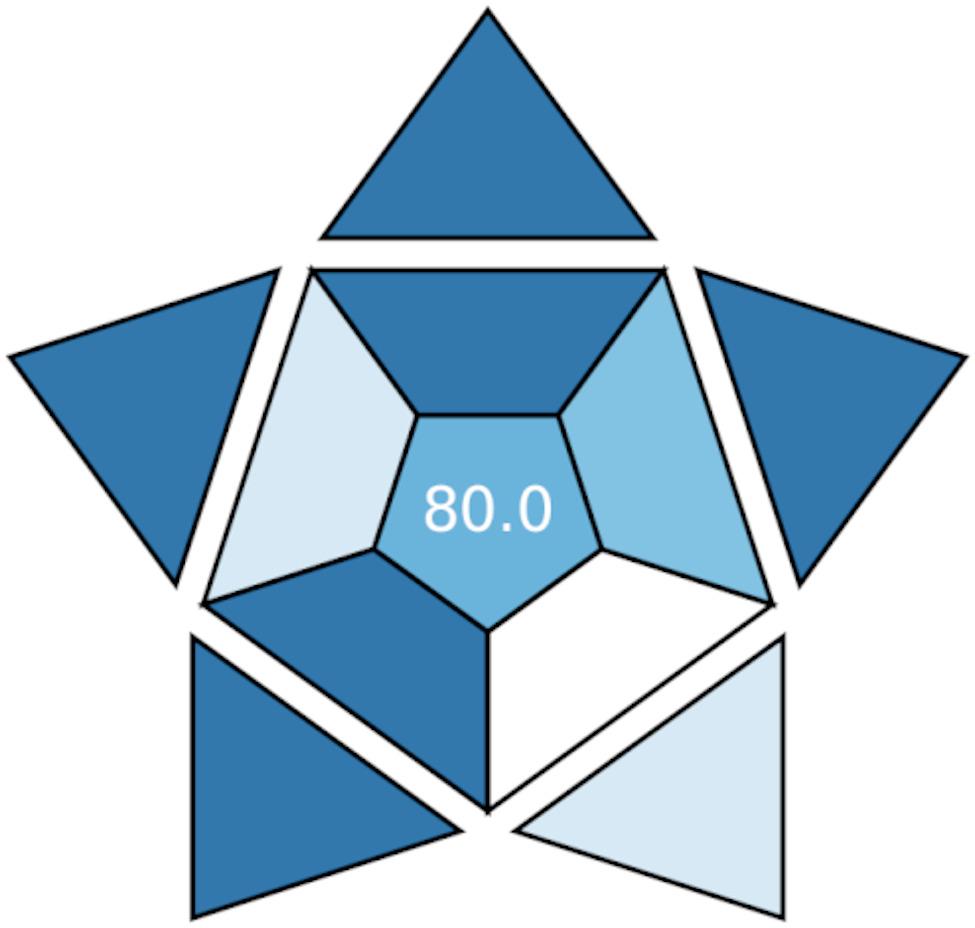
Blueness evaluation of the proposed technique by the BAGI tool

The BAGI tool is a novel metric that evaluates the feasibility – or “blueness” or “practicality” - of an analytical technique, providing both visual and numerical assessments. It uses a color-coded scheme, where white indicates no compliance, Light blue indicates low compliance, blue represents medium, And dark blue signifies high compliance. A numerical BAGI score over 60 is considered indicative of a practical method [47]. As illustrated in Fig. 6, the proposed technique achieved a high score of 80, highlighting its strong practical applicability.

The incorporation of sustainability and greenness assessment tools in electrochemistry is no longer optional but is increasingly recognized as both an ethical responsibility and a scientific imperative. These tools facilitate the development of eco-conscious analytical platforms, encourage sustainable laboratory practices, and align electrochemical research with the global movement toward green innovation and responsible science [48].

## Conclusion

A simple, reliable, and cost-effective potentiometric method was developed for the determination of BNZ·HCl using two types of sensors. These sensors were successfully applied to analyze the drug in its pure form, a pharmaceutical cream, and biological fluids, with accurate results and no interference from the sample matrices.

The coated graphite sensor, in particular, stands out as a practical and eco-friendly alternative to traditional methods. Its ease of use, low cost, and portability make it especially suitable for routine quality control, including in resource-limited settings where BNZ·HCl is commonly used.

While some limitations related to sensor durability and selectivity remain, the method offers a strong foundation for broader use in pharmaceutical testing. Continued improvements in sensor design and maintenance can further enhance its reliability in real-world applications.

## Data Availability

All data generated or analyzed during this study are included in this article and the raw data is available from the corresponding author on reasonable request.
